# Household Water Access, Dietary Diversity and Nutritional Status among Preschoolers in Poor, Rural Areas of Central and Western China

**DOI:** 10.3390/nu14030458

**Published:** 2022-01-20

**Authors:** Yaqing Gao, Jie Sheng, Xiaoyi Mi, Mo Zhou, Siyu Zou, Hong Zhou

**Affiliations:** 1 Department of Maternal and Child Health, School of Public Health/National Health Commission Key Laboratory of Reproductive Health, Peking University, Beijing 100191, China; gaoyaqing@bjmu.edu.cn (Y.G.); 2011210093@bjmu.edu.cn (X.M.); 2111210114@bjmu.edu.cn (M.Z.); zousiyu@pku.edu.cn (S.Z.); 2 Department of Biochemistry and Molecular Biology, School of Basic Medical Sciences, Peking University, Beijing 100191, China; sjgtxy@stu.pku.edu.cn

**Keywords:** household water access, dietary diversity, stunting, anemia, central and western China

## Abstract

Poor child feeding and childhood malnutrition are major public health problems in rural central and western China, with little evidence about their environmental determinants. This study aimed to investigate whether household water access is associated with dietary diversity and nutritional outcomes. We analyzed the cross-sectional data of 3727 children aged 6 to 59 months in rural central and western China, applying multivariate linear and logistic models to estimate the effect of water access on children’s anthropometric indices, hemoglobin, and dietary diversity. We found that unimproved water access was linked to a lower likelihood of achieving dietary diversity (OR = 0.65, 95% CI 0.44 to 0.98, *p* = 0.039); lower height-for-age z-score (β = −0.34, 95% CI −0.49 to −0.19, *p* < 0.001) and hemoglobin concentration (β = −2.78, 95% CI −5.16 to −0.41, *p* = 0.022); higher odds of stunting (OR = 1.50, 95% CI 1.01 to 2.25, *p* = 0.047) and anemia (OR = 1.34, 95% CI 1.02 to 1.77, *p* = 0.037). The associations between water access and nutritional outcomes were not explained by dietary diversity and were stronger in children who did not receive iron supplementation. These findings provide evidence for designing water-based nutritional interventions in China.

## 1. Introduction

Poor childhood nutritional status remains a global concern. A total of 22.0%, 6.7%, and 39.8% of children under 5 years of age were estimated to have stunting, wasting, and anemia globally in 2019, respectively [1,2]. The nutritional status of children under 5 years of age in China has improved substantially, with reductions in the prevalence of stunting (from 9.4% in 2010 to 4.8% in 2017), wasting (from 2.3% in 2010 to 1.9% in 2017), and anemia (from 19.0% in 2010 to 18.8% in 2019) [3,4]. However, geographical and urban-rural disparities in nutritional outcomes remain substantial. In 2013, the prevalence of stunting, wasting, and anemia in rural preschool children were 2.6, 1.8, and 1.2 times higher that in urban children, respectively [3,5]. The central and western provinces have performed worse than the eastern provinces [3,5]. Identifying the modifiable risk factors for poor childhood nutritional outcomes in rural areas of central and western China is imperative for developing appropriate interventions and improving nutrition-related long-term outcomes, including educational achievements, productivity, and overall well-being.

Poor feeding practices has been found to be one of the main causes of child malnutrition and anemia in low- and middle-income countries (LMIC) [6]. Dietary diversity, which has been commonly defined as consuming five or more food groups listed by the World Health Organization (WHO) during the previous day, is a core indicator of feeding practices among infants and young children (6–23 months) [7]. Studies involving children conducted in LMIC showed that consuming more WHO food groups was positively associated with height-for-age z-score (HAZ) [8], and negatively associated with the odds of stunting, wasting, and anemia [9,10]. However, globally, the percentage of children aged 6 to 23 months consuming five or more WHO food groups has remained low (21% in 2010 and 24% in 2020) [11]. A study conducted in rural areas in western China estimated that 44.5% of children aged 6 to 23 months consumed less than four WHO food groups on the day before the survey in 2011 [12].

Most research has focused on the social determinants of dietary diversity and nutritional outcomes, such as parental educational level [13], maternal empowerment [14], and household wealth status [9,13]; however, environmental factors have received little attention. Access to sufficient and safe water, one of the Sustainable Development Goals [15], has been found to be consistently associated with a reduced risk of nutrition-impairing diarrheal disease [16]. However, the relationship between household water access, child feeding practices, nutritional outcomes is less conclusive. For example, a multi-country study found an association between improved water access and lower risk of stunting [17]; however, a study conducted in Nepal failed to identify this association [18]. The association between water access and anemia was not established in multi-country pooled samples, however, it was significant in certain countries (e.g., Laos and Burundi) [19,20]. Furthermore, the extent to which other interventions can mitigate environmental risks is unclear. For example, iron supplementation might decrease the negative impact of unimproved water access on hemoglobin concentrations.

Better water access has also been associated with a higher likelihood of having food that requires a large amount of water for production and preparation, such as fish, fruits, and vegetables in households [21], which may in turn affect children’s dietary diversity. However, there have been few studies that examined the association between household water access and dietary diversity, with only one study conducted in India showed that improved water consumption was associated with the consumption of more food groups [22]. In addition, the mediating effect of dietary diversity in the association between household water access and nutritional outcomes remains unknown. Moreover, to the best of our knowledge, no studies have investigated the association between water access and child nutrition and health using data from China.

**Figure 1:**
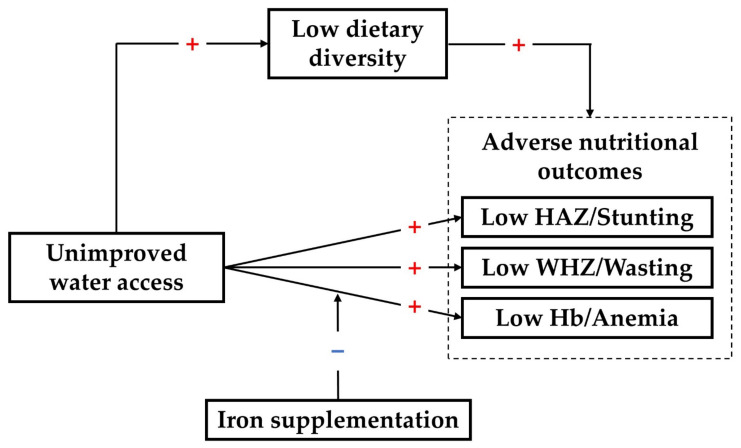
Hypothesized pathways linking water access, dietary diversity, and nutritional status. Unimproved water access may be a risk factor for poor nutritional status (e.g., low height-for-age z-score, stunting, and anemia), dietary diversity may mediate these relationships. Iron supplementation may buffer the negative effect of unimproved water access on the hemoglobin concentration. The red plus sign suggests a positive impact and the blue minus sign suggests a buffering effect. HAZ, height-for-age z-score; WHZ, weight-for-height z-score; Hb, hemoglobin.

In the present study, we used the data collected through a cross-sectional study in 20 rural counties of central and western China to test the hypothesis that unimproved household water access would be associated with adverse nutritional outcomes, as measured by anthropometric indices and hemoglobin concentration, as well as dietary diversity. We also tested whether dietary diversity mediated the associations between household water access and nutritional outcomes. We examined whether the association between household water access and anemia varied depending on whether the child received iron supplementation, which could help design interventions for vulnerable groups (Figure 1).

## 2. Materials and Methods

### 2.1. Data Source

This cross-sectional study was conducted in 20 rural counties in 10 provinces (Hebei, Jiangxi, Henan, Sichuan, Guizhou, Yunnan, Tibet, Qinghai, Ningxia, and Xinjiang) in central and western China between 2016 and 2017, as part of the baseline survey of an early childhood development program funded by United Nations International Children’s Emergency Fund (UNICEF). 

The survey employed a multistage sampling design. First, 15 administrative villages, the lowest administrative units in China, were selected from each county with probability being proportional to village size. Second, in each administrative village, two natural villages, which are naturally formed population clusters, were selected based on the probability proportional to size sampling. Third, 10 households were randomly selected within each natural village for a standardized, face-to-face interview by trained enumerators from universities, UNICEF, and local governments. Information on demographic, housing, child health, and child nutrition was collected from the primary caregiver (the person who knows the conditions of the child best) of the youngest child aged 0 to 59 months within each household. Anthropometric and hemoglobin measures of the child were also collected during the fieldwork. Details of this survey have been described in previous studies [23,24,25].

As children younger than 6 months are considered to be protected against iron deficiency and anemia by iron stores at birth and breastfeeding [26], we excluded children younger than 6 months old in our sample. Therefore, in the analysis of the association between household water access and nutritional outcomes, we restricted our sample to children aged 6 to 59 months.

As the WHO recommended minimum dietary diversity is a proxy for optimal feeding practice for children aged 6 to 23 months [7], we restricted our sample to children aged 6 to 23 months in the analyses that involved dietary variables.

### 2.2. Exposures

We classified household water access based on the indicators for monitoring sustainable water, sanitation and hygiene (WASH) practices developed by WHO and UNICEF [27]. Improved water sources include piped water, tube wells or boreholes, protected springs, protected wells, rainwater, tanker truck, and bottled water. Unimproved water sources include unprotected springs, unprotected wells, and surface water (e.g., lake, river, and pond).

### 2.3. Outcomes

Dietary data based on the caregiver’s recall of food items consumed by the child over a 24-h period preceding the interview were collected through a questionnaire that was used in UNICEF Multiple Indicator Cluster Survey [28]. According to the WHO [7], the food items were assigned to eight food groups (Table 1). We defined a child aged 6 to 23 months as having achieved dietary diversity if the child consumed foods from at least 5 out of 8 food groups during the 24-h period preceding the interview [7].

Stunting and wasting were selected as nutritional status outcomes, which indicate chronic and acute malnutrition, respectively [29]. The height and weight of the child were converted to HAZ and weight-for-height z-score (WHZ) based on the WHO growth reference standard [30]. Stunting is defined by a HAZ below −2, wasting is defined by a WHZ below −2 [29].

Anemia was also selected as a nutritional status outcome. There would be a lower effective hemoglobin count as altitude increases, since the supplies of oxygen become more limited at high altitude. Therefore, an adjustment of the hemoglobin count was made for the children who resided at altitude ≥1000 m above sea level with the following formula [31]:

adjust = −0.32 × (altitude in meters × 0.0033) + 0.22 × (altitude in meters × 0.0033)^2^

adjHb = Hb − adjust, if adjust > 0

Anemia is defined as an altitude-adjusted hemoglobin concentration of <110 g/L for children aged 6–59 months, and can be further classified as moderate to severe anemia if the hemoglobin concentration is below 100 g/L [32].

### 2.4. A Priori Confounding Variables

The demographic covariates included: age (months), sex, birth order (first-born/later-born), primary caregiver of the child (mother/father/grandmother/grandfather/others); whether the child was left-behind (yes/no), education level (illiterate/primary school/middle school/high school/college and above) and smoking status (currently smoking/not smoking) of the primary caregiver, and household wealth quintile. To reduce household environmental confounding, we also controlled for household sanitation facilities (improved/unimproved/unknown) and whether the primary caregiver clean his/her hands using soap (yes/no/unknown).

The left-behind child was defined as one or more parents leaving the place in which their children were living to find work. Improved sanitation facilities include flush/pour flush to piped sewer system, septic tanks, and pit latrines. Unimproved sanitation facilities include pit latrines without a slab or platform, hanging latrines, and bucket latrines [27]. The household wealth quintile was constructed following the method recommended by the World Food Programme [33,34]. The questionnaire collected data on household’s ownership of a list of assets. First, we selected the assets items that were owned by >5% and <95% of all households, which included: car, motorcycle, bicycle, refrigerator, landline telephone, computer with internet access, cable television, mobile telephone with internet access, and radio. Then, we calculated the wealth index for each household according to the first component of the principal component analysis of the ownership of selected assets. Finally, we divided the entire household population into quintiles from the poorest (Wealth Quintile 1) to the richest (Wealth Quintile 5) based on household wealth index.

### 2.5. Statistical Analysis

We applied logistic regression models to investigate the associations of household water access with each of the 8 food groups and dietary diversity among children aged 6 to 23 months. To determine the relationship between household water access and nutritional status of children aged 6 to 59 months, linear regression models were used for continuous outcomes (HAZ, WHZ, and hemoglobin concentration), and logistic regression was performed for binary outcomes (stunting, wasting, anemia, and moderate to severe anemia). We first estimated the crude effects from the baseline model that did not adjusted for any covariate, and then estimated the adjusted effects from models that adjusted for all a priori confounding variables.

For children aged 6 to 23 months, we additionally included dietary diversity in the models to examine whether dietary diversity is a plausible explanation for the association between household water access and nutritional outcomes. The potential interaction between iron supplementation and household water access was explored by adding interaction terms to the models and examining multiplicative scales. We also tested for effect modification by analyses stratified by iron supplementation. The receipt of iron supplementation was defined as a having consumed iron supplements or iron-fortified food during the 24-h period preceding the interview.

For all models, standard errors were clustered at the village level to account for the neighborhood-level confounding. Statistical significance was set at *p*-value less than 0.05. All data were analyzed using R 3.6.1 (R Foundation, Vienna, Austria).

## 3. Results

### 3.1. Population Characteristics

The study included 3727 children aged 6 to 59 months. Children’s mean age was 28.0 months, and 45.3% were female (Table 2). On average, only two-thirds (67.0%) of primary caregivers were mothers of the children, and 46.2% of children were left-behind. The primary caregivers of 17.9% of children were illiterate.

Approximately 86.6% of households had improved water access. Compared with children who lived in households with improved water access, those whose households had unimproved water access were poorer, more likely to have older siblings and be left-behind and had primary caregivers who were less educated, more likely to smoke, and less likely to wash their hands with soap.

### 3.2. Association between Household Water Access and Dietary Diversity

Dietary diversity was achieved in 49.8% of the 1646 children aged 6 to 23 months included in the study population (Table 3). During the 24-h period preceding the interview, the most commonly food group consumed by children was grains, roots, and tubers (89.4%), followed by Vitamin A-rich fruits and vegetables (71.3%) and dairy products (60.1%). Foods in the “legumes, nuts and seeds” group were consumed by the least number of the children (22.3%).

The proportion of children who achieved dietary diversity was higher in households with improved water access than in those with unimproved water access (55.5% vs. 21.7%), which corresponds to a 78% reduction in the odds of achieving dietary diversity for children with unimproved water access. After controlling for covariates, unimproved water was associated with a 35% reduction in the odds of achieving dietary diversity (odds ratio [OR] = 0.65, 95% confidence interval [CI] 0.44 to 0.98, *p* = 0.039). Similar associations were found for breast milk (OR = 0.56, 95% CI 0.33 to 0.95, *p* = 0.032), vitamin A-rich fruits and vegetables (OR = 0.51, 95% CI 0.35 to 0.75, *p* = 0.001), and other fruits and vegetables (OR = 0.55, 95% CI 0.36 to 0.83, *p* = 0.004). 

### 3.3. Association between Household Water Access and Nutritional Outcomes

For children aged 6 to 59 months, the mean HAZ, WHZ and hemoglobin concentration was −0.42 (standard deviation [SD] 1.21), 0.43 (1.13), and 109.97 (16.62) g/L, respectively (Table 4). The most prevalent adverse nutritional outcome was anemia, with 44.2% of children classified as anemic and 23.1% of children experienced moderate to severe anemia. Stunting (8.5%) and wasting (2.0%) were less prevalent.

In the crude model, children had lower HAZ, WHZ, and hemoglobin concentration, as well as higher odds of being stunted and anemic if they lived in households with unimproved water access. After controlling for covariates, in households with unimproved water access, the HAZ (β = −0.34, 95% CI −0.49 to −0.19, *p* < 0.001) and hemoglobin concentration (β = −2.78, 95% CI −5.16 to −0.41, *p* = 0.022) were significantly lower, which corresponds to a 50% increase in the odds of stunting (OR = 1.50, 95% CI 1.01 to 2.25, *p* = 0.047) and a 34% increase in the odds of anemia (OR = 1.34, 95% CI 1.02 to 1.77, *p* = 0.037).

### 3.4. The Mediating Effect of Dietary Diversity

**Figure 2:**
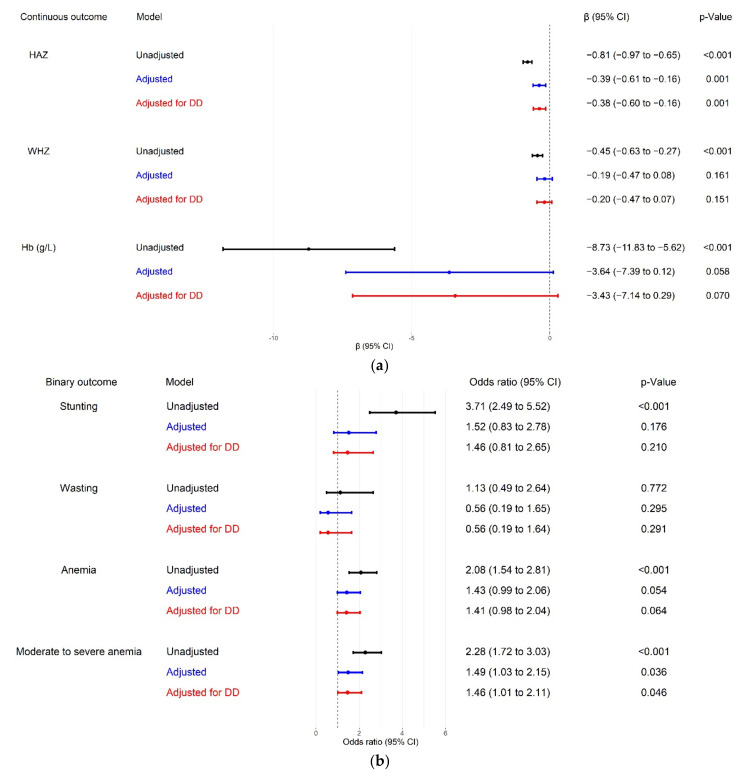
Associations between household water access and nutritional outcomes in children aged 6 to 23 months. In “adjusted” models, we adjusted for all a priori confounding variables. In “adjusted for DD” models, we adjusted for all a priori confounding variables and dietary diversity. (**a**) Associations between household water access and continuous nutritional outcomes; (**b**) associations between household water access and binary nutritional outcomes. HAZ, height-for-age z-score; WHZ, weight-for-height z-score; Hb, hemoglobin; DD, dietary diversity; CI, confidence interval.

When we restricted the analysis to children aged 6 to 23 months, the crude models showed an association of unimproved water access with all three continuous nutritional outcomes, as well as three binary outcomes, except for wasting (Figure 2). After adjusting for covariates, statistical significance persisted for the association of unimproved water access with HAZ (β = −0.39, 95% CI −0.61 to −0.16, *p* = 0.001) and moderate to severe anemia (OR = 1.49, 95% CI 1.03 to 2.15, *p* = 0.036).

After additional adjustment for dietary diversity, the pattern of association between HAZ and moderate-to-severe anemia was largely unchanged, with an estimate of β increasing from −0.39 to −0.38 for HAZ, whereas that of the OR decreasing from 1.49 to 1.46 for moderate to severe anemia. 

### 3.5. The Moderating Effect of Iron Supplementation

**Figure 3:**
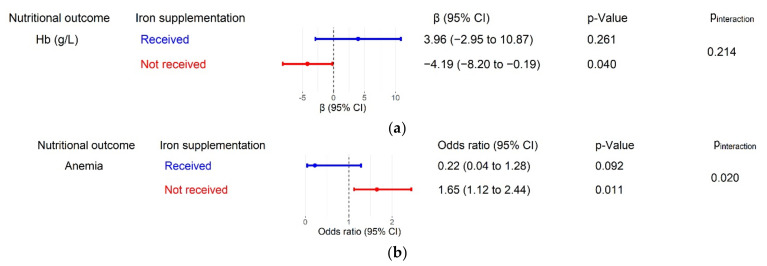
Stratified associations of household water access with anemia in children aged 6 to 23 months, adjusted for all a priori confounding variables. Pineraction is the coefficient of the interaction term between iron supplementation and household water access (i.e., *p*-value for multiplicative interaction). (**a**) Associations between household water access and hemoglobin concentration stratified by iron supplementation status; (**b**) associations between household water access and anemia stratified by iron supplementation status. Hb, hemoglobin; CI, confidence interval.

The association of household water access with anemia-related outcomes was stronger among children who did not receive iron supplementation within 24 h before the survey (Figure 3). Unimproved water access was associated with lower hemoglobin concentration in children who did not receive iron supplementation (β = −4.19, 95% CI −8.20 to −0.19, *p* = 0.040), but not in children who received it (β = −3.96, 95% CI −2.95 to 10.87, *p* = 0.261), although the difference was not statistically significant (*p* for interaction = 0.214). The association between unimproved water access and anemia was also detected in children who did not receive iron supplementation (OR = 1.65, 95% CI 1.12 to 2.44, *p* = 0.011) but not in children who received it (OR = 0.22, 95% CI 0.04 to 1.28, *p* = 0.092). The difference between the two groups was statistically significant (*p* for interaction = 0.020).

## 4. Discussion

### 4.1. Principal Findings

According to our literature search, the present study is the first to examine the association between household water access and children’s nutrition-related outcomes in China. We found that unimproved water access was associated with 35% lower odds of achieving dietary diversity in children aged 6 to 23 months, which was mainly reflected by lower odds of consuming breast milk, vegetables, and fruits. We also found that in children aged 6 to 59 months, unimproved water access was associated with low HAZ and hemoglobin concentration, as well as a 50% and 34% increase in the odds of stunting and anemia, respectively. Wasting or WHZ was not associated with household water access. Meanwhile, we found little evidence that the associations between water access and nutritional outcomes were explained by dietary diversity. Statistically significant associations of unimproved water access with hemoglobin concentration and anemia were only observed in children who did not receive iron supplementation.

### 4.2. Comparison with Other Studies

Our findings are consistent with those of a study conducted in India that showed that piped or bottled water was associated with higher odds of access to dietary diversity than protected/unprotected springs and surface water [22]. Several possible mechanisms are behind this association. First, several foods require large quantities of water for growth (e.g., irrigation) and processing (e.g., wash-down). In water-stressed environments, diet might be limited to few foods that require less of water during production, including beans and wheat, rather than fresh fruits and vegetables [21]. Second, unimproved water access might have negative economic consequences, including increased time costs for fetching water from distant sources and medical expenditure for treating illness due to contaminated water from unprotected wells. This might decrease budgets for adequate and diverse food for a family [35,36]. Notably, in China, eastern provinces have a higher production output of vegetables and fruits and have lower prices than the central and western provinces [37]. Therefore, the impact of limited water access on the purchase of vegetables and fruits could be more pronounced in central and western China, as found in the present study. Third, anxiety and depression may develop because of the difficulties in acquiring safe water [36]. Poor maternal mental status, in turn, is associated with reduced dietary diversity in the offspring [38,39]. Fourth, unimproved water access might increase the risk of maternal exposure to intimate partner violence [40], which has been established as a risk factor for inadequate complementary feeding and early termination of exclusive breastfeeding [41,42]. 

Our findings also add to the body of literature documenting the association between household water access and nutritional outcomes in LMIC. Evidence from a merged data set covering children aged 0 to 59 years in 70 LMIC suggests that unimproved household water sources elevated children’s risk of stunting [17]. A study conducted in children aged 3 to 36 months in eight LMIC found an association between improved water access and increased HAZ only in children living in rural areas [43], which is the same setting as that of the present study. Therefore, rural children might be more susceptible to the effects of inadequate water access on growth than urban children, potentially owing to poor hygiene infrastructure and healthcare services in rural areas, whose effects on stunting could be exacerbated by unimproved water access [44]. Indeed, a study conducted in Nepal with 91.4% of sample households located in wards with developed infrastructure (paved roads, hospitals, and bazaar) did not find an association between protected water sources and a decreased risk of stunting in children aged 6 to 59 months [18]. 

We found that unimproved water access was associated with lower hemoglobin concentration and higher risk of anemia in children aged 6 to 59 months, which is consistent with the results of another study conducted in Tanzania that showed an increase in the hemoglobin concentrations in children whose households had tap water compared with those who used public wells [45]. A multi-country study showed heterogeneity in the associations between household water sources and anemia across countries. In 12 of the 40 countries included in the study, children using surface water had higher odds of anemia than those with improved water (piped water/protected springs). However, surface water consumption was a protective factor for anemia in five countries, with the strongest effect observed in India. The seemingly paradoxical finding could be due to the presence of high levels of natural iron in the surface water in India [19]. A study of rural Filipino children aged 12 to 71 months also found that the quality of surface water was an independent factor affecting hemoglobin concentration, with higher hemoglobin concentration in children using spring water from Dalaguete than that in children using surface water from Barili, where the water was more likely to be contaminated [46]. The effect of the quality of surface water should be relatively small in our study, as the surface water is very limited in western China [47].

There are two possible mechanisms that can explain the relationship between household water access and child nutritional outcomes. First, the mediating role of dietary diversity was detected in a study of Indian children aged 6 to 23 months [22], which was not observed in our study. This may be partly due to the transient nature of the 24-h recall measure of dietary intake, which in turn could non-differentially misclassify the children’s status of dietary diversity and bias the effect of the mediator toward the null [48]. Second, water insecurity might result in long-term exposure to pathogens, leading to chronic systemic immune activation, which in turn suppresses nutrient absorption and growth hormone production, as well as increases nutrient losses [49]. In our study, household water access was not associated with WHZ or wasting, which indicates that the effect of unimproved water might have an impact on long-term nutritional status and is not reflected in acute malnutrition. Meanwhile, we observed that point estimations of the effects of unimproved water access on hemoglobin concentration and anemia were higher, although not always statistically significant, in children who did not receive iron supplementation. This suggests that fortified food with micronutrients, including iron, might counteract nutrient loss and malabsorption resulting from exposure to an unhygienic environment. 

### 4.3. Strength and Limitations

In addition to the limitation of the measure of children’s diet discussed above, this study has other limitations. First, the associations of household water access with dietary diversity and nutritional outcomes, as well as the results of the mediation analysis are yet to be causally established owing to the cross-sectional nature of the data. Second, the analysis did not include data on water pollution or the process of safe water handling in the household, resulting in possible non-differential misclassification of exposure. Third, no information on parental nutritional status was collected, which is an important factor that may affect children’s nutritional outcomes [9]. However, we adjusted for multiple indicators of household socio-economic status (e.g., household wealth status and caregiver’s educational level), which may partly control the parental nutritional and health status. Finally, data were obtained from poor rural counties in western China with inadequate health literacy and a heavy burden of child malnutrition [3,50]. Therefore, generalizing our findings to the entire country should be performed with caution.

The strength of this study is that it is the first to investigate the role of household water access in multiple nutrition-related outcomes in China. Current WHO and UNICEF guidelines have underscored the importance of implementing the “water, sanitation, and hygiene (WASH)” interventions in LMIC [51]. However, the evidence base for WASH interventions remains limited in China due to the lack of observational studies examining the environmental determinants of child nutrition. Moreover, WASH interventions primarily focused on the nutritional outcomes (e.g., HAZ and hemoglobin concentration) [52,53], with little attention to intermediate outcomes, such as dietary diversity. Further, few WASH interventions have quantified the extent of the contribution made by each component of WASH. The present study focused on household water access as a single aspect by controlling the household sanitation facilities and caregivers’ handwashing practices in the models and, therefore, provides evidence for selecting program content and designing cost-effective child nutrition interventions in China. 

### 4.4. Implications for Future Research

Future work to establish causality and assess the underlying mechanisms of the association between household water access and nutritional outcomes is crucial for designing water-based interventions, which may help to improve feeding practices and reduce the burden of malnutrition in children in central and western China.

Poor nutritional status during early development may have long lasting effects on physical and mental health. For example, poor dietary may harm not only children’s physical health but also their developing brain [54]. Possible long-term consequences of early childhood anemia include bone disease, growth impairment and pubertal delay, decreased motor activity, impaired cognitive development, and altered cardiac function [55]. Stunting also has adverse long-term consequences such as impaired cognition development, poor education performance, and low adult productivity [56]. Further investigation on the prospective associations between household water access in early life and these negative long-term consequences and whether nutritional status mediated the effects is warranted.

### 4.5. Policy Implications 

In China, rural water infrastructure is managed by the Central Government (National Development and Reform Commission, Ministry of Water Resources, National Health Commission, Ministry of Ecology and Environment, and Ministry of Finance) and the local governments [57]. Generally, operation and maintenance costs of rural piped water supply projects are shared by the Central Government, local government, and users (rural residents) [57]. The investment policies vary across regions with different economic conditions. In recent years, users in the poor regions such as the central and western China are expected to finance a substantially smaller proportion of the costs with the support from the state poverty alleviation funds [58]. Meanwhile, pilot rural water supply projects assisted by non-governmental organizations have been implemented, which might also be scalable solutions to alleviate the fiscal burden of the users [59]. The prevalence of houses with unimproved water access decreased from 13% in 2015 to 9% in 2020 in rural China [60]. However, unacceptable inequalities persist, with 2% of the urban inhabitants living in houses with unimproved water access in 2020 [60]. 

Based on the experience from previous projects, we proposed four levers for change that could lead to sustainable clean water supply in rural China according to the model of change developed by the United Nations for the Sustainable Development Goals [61]: (1) establishing clear jurisdictions over water management in the central and local governments; (2) prioritizing public finances in support of water supply projects and the related research; (3) producing scientific evidence of long-term benefits of improved water access; (4) raising public awareness about clean water access. However, deploying these levers to bring about the systematic transformations in rural water access may take time. Therefore, in the interim, measures could be implemented by communities and families to buffer against the negative impact of unimproved water access on nutrition-related outcomes. For example, community health professionals could identify children with malnutrition early through targeted screening of children living in houses without improved water access; caregivers of these children should be guided to increase compliance towards the public health programs, such as the iron supplementation program, and the “Ying Yang Bao”, a large-scale complementary food fortification program targeting rural children aged 6 to 23 months launched by the Chinese Government [62]. 

## 5. Conclusions

To the best of our knowledge, this study is the first to demonstrate the direct associations of unimproved water access in China, with lower odds of dietary diversity in children aged 6 to 23 months, along with lower HAZ and hemoglobin concentration and higher risks of stunting and anemia in children aged 6 to 59 months. Specifically, the present study highlighted that the association between water access and anemia is most apparent in children who did not receive iron supplementation. 

## Figures and Tables

**Table 1:** Questionnaire food items mapped to food groups created by WHO.

WHO Food Groups	Food Items in the Questionnaire
Breast milk	Breast milk
Grains, roots and tubers	Bread, rice, noodles, white potatoes, white yams, manioc, cassava
Legumes, nuts and seeds	Beans, peas, lentils, nuts
Dairy products	Infant formula, milk from animals (fresh, tinned or powdered), yogurt, hard or soft cheese
Flesh foods	Liver, kidney, heart, beef, pork, lamb, goat, chicken, duck, fresh or dried fish, shellfish, shrimps
Eggs	Eggs
Vitamin A-rich fruits and vegetables	Pumpkin, carrots, sweet red peppers, squash or sweet potatoes that are yellow or orange inside; dark green leafy vegetables such as spinach; ripe mangoes, ripe papayas, tomato, Chinese hawthorn, persimmon
Other fruits and vegetables	Any other fruits and vegetables

WHO, World Health Organization.

**Table 2:** Sample characteristics stratified by household water access.

Characteristics	Total Sample (*N* = 3727) ^2^	Household Water Access	
		Improved (*N* = 3228, 86.6%)	Unimproved (*N* = 499, 13.4%)
Age (months) ^1^	28.0 (14.8)	28.5 (14.9)	24.4 (13.9)
Sex			
Female	1687 (45.3)	1453 (45.0)	234 (46.9)
Male	2040 (54.7)	1775 (55.0)	265 (53.1)
Birth order			
First-born	1493 (40.1)	1346 (41.7)	147 (29.5)
Later-born	2234 (59.9)	1882 (58.3)	352 (70.5)
Primary caregiver			
Mother	2496 (67.0)	2168 (67.2)	328 (65.7)
Father	290 (7.8)	252 (7.8)	38 (7.6)
Grandmother	716 (19.2)	611 (18.9)	105 (21.0)
Grandfather	185 (5.0)	167 (5.2)	18 (3.6)
Others	40 (1.1)	30 (0.9)	10 (2.0)
Left-behind			
Yes	1721 (46.2)	1442 (44.7)	279 (55.9)
No	2006 (53.8)	1786 (55.3)	220 (44.1)
Education level			
Illiterate	666 (17.9)	380 (11.8)	286 (57.3)
Primary school	844 (22.6)	722 (22.4)	122 (24.4)
Middle school	1570 (42.1)	1497 (46.4)	73 (14.6)
High school	415 (11.1)	404 (12.5)	11 (2.2)
College and above	232 (6.2)	225 (7.0)	7 (1.4)
Currently smoking			
Yes	300 (8.0)	225 (7.0)	75 (15.0)
No	3427 (92.0)	3003 (93.0)	424 (85.0)
Household wealth quintile ^3^			
1	730 (19.6)	400 (12.4)	330 (66.1)
2	735 (19.7)	639 (19.8)	96 (19.2)
3	745 (20.0)	709 (22.0)	36 (7.2)
4	752 (20.2)	733 (22.7)	19 (3.8)
5	765 (20.5)	747 (23.1)	18 (3.6)
Household sanitation facilities			
Improved	2561 (68.7)	2171 (67.3)	390 (78.2)
Unimproved	1122 (30.1)	1038 (32.2)	84 (16.8)
Unknown	44 (1.2)	19 (0.6)	25 (5.0)
Handwashing with soap			
Yes	2456 (65.9)	2269 (70.3)	187 (37.5)
No	1207 (32.4)	937 (29.0)	270 (54.1)
Unknown	64 (1.7)	22 (0.7)	42 (8.4)

^1^ Estimates are mean (standard deviation) for age, and *N* (%) for other characteristics. ^2^ Number of children aged 6 to 59 months whose dietary, anthropometric and hemoglobin data were available. ^3^ Quintile 1, poorest; Quintile 2, poorer; Quintile 3, middle; Quintile 4, richer; Quintile 5, richest.

**Table 3:** Associations of household water access with food groups and dietary diversity in children aged 6 to 23 months.

Food Groups	Total Sample (*N* = 1646) ^1^	Household Water Access		Unadjusted		Adjusted ^3^	
		Improved (*N* = 1369, 83.2%) ^2^	Unimproved (*N* = 277, 16.8%) ^2^	OR (95% CI)	*p*-Value	OR (95% CI)	*p*-Value
Breast milk	596 (38.9)	495 (38.8)	101 (39.1)	1.02 (0.72 to 1.43)	0.932	0.56 (0.33 to 0.95)	0.032
Grains, roots and tubers	1472 (89.4)	1235 (90.2)	237 (85.6)	0.64 (0.42 to 0.99)	0.043	0.90 (0.53 to 1.53)	0.695
Legumes, nuts and seeds	366 (22.3)	339 (24.8)	27 (9.8)	0.33 (0.20 to 0.55)	<0.001	0.71 (0.39 to 1.28)	0.252
Dairy products	989 (60.1)	879 (64.2)	110 (39.7)	0.37 (0.27 to 0.51)	<0.001	0.98 (0.62 to 1.53)	0.913
Flesh foods	944 (57.4)	822 (60.0)	122 (44.0)	0.52 (0.39 to 0.71)	<0.001	0.89 (0.62 to 1.30)	0.561
Eggs	777 (47.3)	691 (50.5)	86 (31.0)	0.44 (0.31 to 0.62)	<0.001	1.08 (0.72 to 1.64)	0.709
Vitamin A-rich fruits and vegetables	1174 (71.3)	1040 (76.0)	134 (48.4)	0.30 (0.22 to 0.40)	<0.001	0.51 (0.35 to 0.75)	0.001
Other fruits and vegetables	834 (50.9)	768 (56.2)	66 (24.1)	0.25 (0.17 to 0.36)	<0.001	0.55 (0.36 to 0.83)	0.004
Dietary diversity	820 (49.8)	760 (55.5)	60 (21.7)	0.22 (0.16 to 0.31)	<0.001	0.65 (0.44 to 0.98)	0.039

OR, odds ratio; CI, confidence interval. ^1^ Number of children aged 6 to 23 months whose dietary, anthropometric and hemoglobin data were available. Estimates are *N* (%). ^2^ Estimates are *N* (%). ^3^ Adjusted for all a priori confounding variables.

**Table 4:** Associations between household water access and nutritional outcomes in children aged 6 to 59 months.

Nutritional Outcomes	Total Sample (*N* = 3727) ^1^	Improved Water Access (*N* = 3228, 86.6%)	Unimproved Water Access (*N* = 499, 13.4%)	Unadjusted		Adjusted ^2^	
Continuous Outcomes	Mean (SD)	Mean (SD)	Mean (SD)	β (95% CI)	*p*-Value	β (95% CI)	*p*-Value
HAZ	−0.42 (1.21)	−0.33 (1.18)	−1.06 (1.21)	−0.73 (−0.86 to −0.61)	<0.001	−0.34 (−0.49 to −0.19)	<0.001
WHZ	0.43 (1.13)	0.46 (1.11)	0.22 (1.21)	−0.25 (−0.37 to −0.13)	<0.001	−0.11 (−0.27 to 0.06)	0.205
Hb concentration (g/L)	109.97 (16.62)	111.08 (15.99)	102.79 (18.70)	−8.29 (−10.59 to −5.99)	<0.001	−2.78 (−5.16 to −0.41)	0.022
Binary Outcomes	*N* (%)	*N* (%)	*N* (%)	OR (95% CI)	*p*-Value	OR (95% CI)	*p*-Value
Stunting	317 (8.5)	216 (6.7)	101 (20.2)	3.54 (2.69 to 4.66)	<0.001	1.50 (1.01 to 2.25)	0.047
Wasting	74 (2.0)	62 (1.9)	12 (2.4)	1.26 (0.65 to 2.43)	0.495	0.56 (0.24 to 1.30)	0.174
Anemia	1649 (44.2)	1349 (41.8)	300 (60.1)	2.10 (1.67 to 2.64)	<0.001	1.34 (1.02 to 1.77)	0.037
Moderate to severe anemia	860 (23.1)	674 (20.9)	186 (37.3)	2.25 (1.78 to 2.85)	<0.001	1.25 (0.94 to 1.67)	0.128

HAZ, height-for-age z-score; WHZ, weight-for-height z-score; Hb, hemoglobin; SD, standard deviation; OR, odds ratio; CI, confidence interval. ^1^ Number of children aged 6 to 59 months whose dietary, anthropometric and hemoglobin data were available. Estimates are *N* (%). ^2^ Adjusted for all a priori confounding variables.

## Data Availability

The data are available on request.
